# Altered Insulin/Insulin-Like Growth Factor Signaling in a Comorbid Rat model of Ischemia and β-Amyloid Toxicity

**DOI:** 10.1038/s41598-018-22985-4

**Published:** 2018-03-23

**Authors:** Zareen Amtul, David J. Hill, Edith J. Arany, David F. Cechetto

**Affiliations:** 1 Department of Anatomy and Cell Biology, University of Western Ontario, London, N6A 5C1 Canada; 2 Departments of Medicine, Physiology and Pharmacology, and Pediatrics, University of Western Ontario, London, N6A 5C1 Canada; 3 Lawson Health Research Institute, London, Ontario, N6A 4V2 Canada; 4 Department of Pathology and Laboratory Medicine, University of Western Ontario, London, N6A 5C1 Canada

## Abstract

Ischemic stroke and diabetes are vascular risk factors for the development of impaired memory such as dementia and/or Alzheimer’s disease. Clinical studies have demonstrated that minor striatal ischemic lesions in combination with β-amyloid (Aβ) load are critical in generating cognitive deficits. These cognitive deficits are likely to be associated with impaired insulin signaling. In this study, we examined the histological presence of insulin-like growth factor-I (IGF-1) and insulin receptor substrate (IRS-1) in anatomically distinct brain circuits compared with morphological brain damage in a co-morbid rat model of striatal ischemia (ET1) and Aβ toxicity. The results demonstrated a rapid increase in the presence of IGF-1 and IRS-1 immunoreactive cells in Aβ + ET1 rats, mainly in the ipsilateral striatum and distant regions with synaptic links to the striatal lesion. These regions included subcortical white matter, motor cortex, thalamus, dentate gyrus, septohippocampal nucleus, periventricular region and horizontal diagonal band of Broca in the basal forebrain. The alteration in IGF-1 and IRS-1 presence induced by ET1 or Aβ rats alone was not severe enough to affect the entire brain circuit. Understanding the causal or etiologic interaction between insulin and IGF signaling and co-morbidity after ischemia and Aβ toxicity will help design more effective therapeutics.

## Introduction

Vascular cognitive impairment (VCI) refers to cognitive impairment that is associated with, or caused by, vascular factors^1,2^. In the elderly VCI risk factors occur in the presence of high levels of amyloid. Clinical studies have demonstrated that minor striatal ischemic lesions are very critical in generating cognitive deficits in combination with β-amyloid (Aβ) load^3^.

Clinical investigations have clearly established an interaction between Alzheimer’s disease (AD) and ischemia^3^. In this regard, we have shown using the present co-morbid striatal ischemia and Aβ toxicity rat model, the presence of high levels of endogenous amyloid, amyloid precursor protein (APP), microgliosis, astrocytosis and increased ischemia size in cortical, striatal and hippocampal regions that eventually led to cognitive deficits^4–6^. We have also provided mechanistic insight into the correlation between hippocampal pathogenesis, progenitor cells and cognitive impairment^6^ in co-morbid neuropathologies.

The clinical literature presents a compelling argument for impaired insulin signaling in the vulnerable brains, such as those developing Alzheimer’s type pathologies^7,8^ or ischemia. Diabetes is considered to be one of the risk factors for senile dementia of the Alzheimer’s type^9,10^. There is a growing interest in understanding the status and function of insulin signaling in AD brain, especially since defects in insulin, insulin receptor (IR), insulin receptor substrate (IRS-1)^11^ and, increased insulin-like growth factor-I (IGF-1) levels in astrocytes^12,13^ as well as decreased IGF-1/IGF-II levels^14^ have been reported in AD. Impaired insulin signaling is also thought to be a factor contributing to neuronal degeneration in AD by impairing the cellular clearance of neurotoxic oligomeric Aβ^11^, promoting amyloid generation and eventually Aβ plaque burden^14,15^. AD has also been hypothesized as a brain specific ‘type 3’ diabetes^14,16^. IRS-1 interacts with many proteins involved in neurodegenerative pathways^17^. Similarly, IGF-1 signaling mediated through PI3-kinase/Akt is a key pathway for neuronal survival and growth, and has been demonstrated to be involved in the survival of neurons in ischemic brain and spinal cord injury models and in several types of neuronal insults^18–23^. Moreover, behavioral outcomes after traumatic brain injury have been improved by IGF-1 administration^24^.

Although studies in patients clearly indicate impaired insulin signaling in both AD and stroke, there has been little investigation of insulin or IGF signaling as a consequence of striatal ischemia and Aβ toxicity, as well as the etiologic link between ischemia and AD. Thus, it is critical to investigate insulin signaling in the brain of comorbid animal models of vascular risk factor such as ischemia in the presence of elevated levels of brain amyloid (adult onset sporadic AD model). It will further help to examine important clinical conditions of co-existing morbidities related to VCI and to accurately replicate the metabolic and cellular conditions of the human diseases where these conditions coexist in the elderly.

Hence, in the present investigation we examined changes in the expression of IGF-1 and IRS-1 in the combined model of cerebral ischemia and Aβ toxicity. In particular, this study focused on the potential synergism that may account for the clinical findings to possibly assist in an early diagnosis of the brain at risk for AD.

## Results

**Figure 1:**
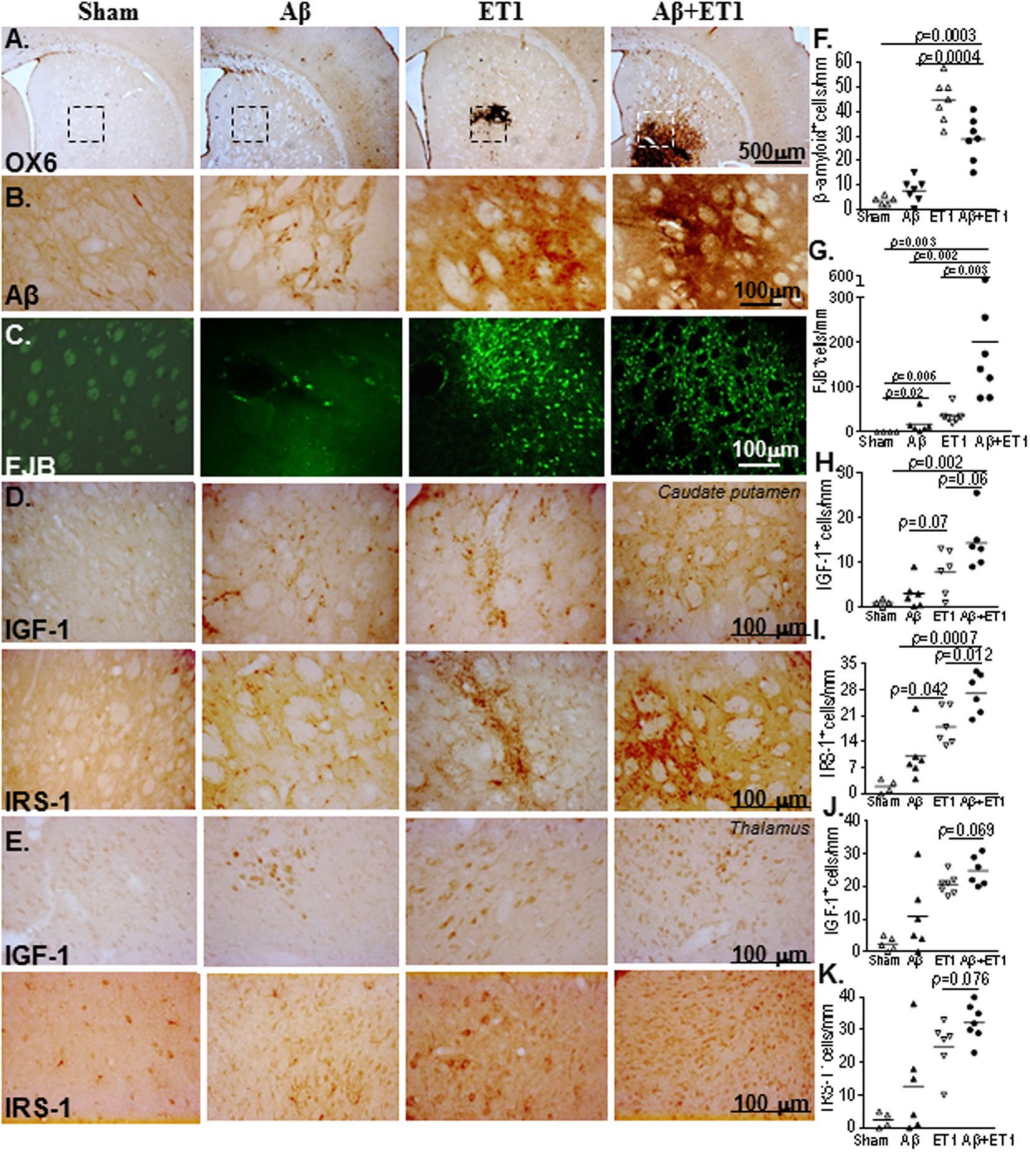
Striatum and thalamus: Low resolution images at bregma levels +0.48 mm show cerebral injury in the striatal lesion core of ET1 and Aβ + ET1 rats. (**A**) The dotted rectangles indicate the region of the high magnification images in **B**,**C**,**D** and **E**. High resolution immunostaining indicates staining for striatal expression of APP fragments including β-amyloid (**B**) and FJB degeneration (**C**) as well as IGF-1 and IRS-1 in the ipsilateral striatum (**D**) and thalamus (**E**) of sham, Aβ, ET1 and Aβ+ET1 rats Plots show quantitative assessment of β-amyloid (**F**), FJB (**G**), IGF-1 and IRS-1 staining in the striatum (**H** and **I**) and thalamus (**J** and **K**) of sham, Aβ, ET1 and Aβ+ET1 rats, respectively. (**B,C,F** and **G** are courtesy from^5)^.

The Aβ + ET1-treated rat model was shown by us^5,6,25^ to exhibit several hallmark features indicative of a degenerating brain including, but not limited to, the significantly increased number of OX6 (Fig. 1A), amyloid (Fig. 1B)^5^ and fluorojade B (Fig. 1C)^5^ positive degenerating cells compared to the sham rats. In the current investigation, increased IGF-1 and IRS-1 staining was mostly prominent in the striatum, thalamus, cortex, subcortical white matter, hippocampus and septohippocampus of ET1 and Aβ + ET1 rats. Aβ toxicity showed an effect on IGF-1 and IRS-1 presence, it was significantly altered in various brain regions such as dentate gyrus and septohippocampal nucleus as mentioned below. Moreover, an increased number of Aβ-stained cells in ET1 rats compared to the Aβ + ET1 rats (Fig. 1B) that from morphology and distribution pattern appear to be neurons, hints towards the relatively higher neuronal loss in Aβ + ET1 rats, as determined by FJB staining.

### Striatum and thalamus

The majority of the IGF-1 or IRS-1 positive cells found in the caudate putamen (Fig. 1D,H,I) and thalamus (Fig. 1E,J,K) from morphology and distribution look like neurons. Neurons have already been reported to express IRS-1^26,27^. These cells were mostly concentrated in the ipsilateral dorso-medial striatum and ventral posteromedial (VPM) and ventral posterolateral (VPL) nuclei of ET1 and Aβ + ET1 rats. In the caudate putamen Aβ + ET1 rats demonstrated significantly more numbers of IGF-1 (*p* = 0.002) and IRS-1 (*p* = 0.0007) positive cells compared to the Aβ rats. In the thalamus of ET1 and Aβ + ET1 rats, IGF-1 and IRS-1 positive neurons appeared to have deficient dendritic and axonal branching than the sham rats (Fig. 1C).

### Cortex and subcortical white matter

**Figure 2:**
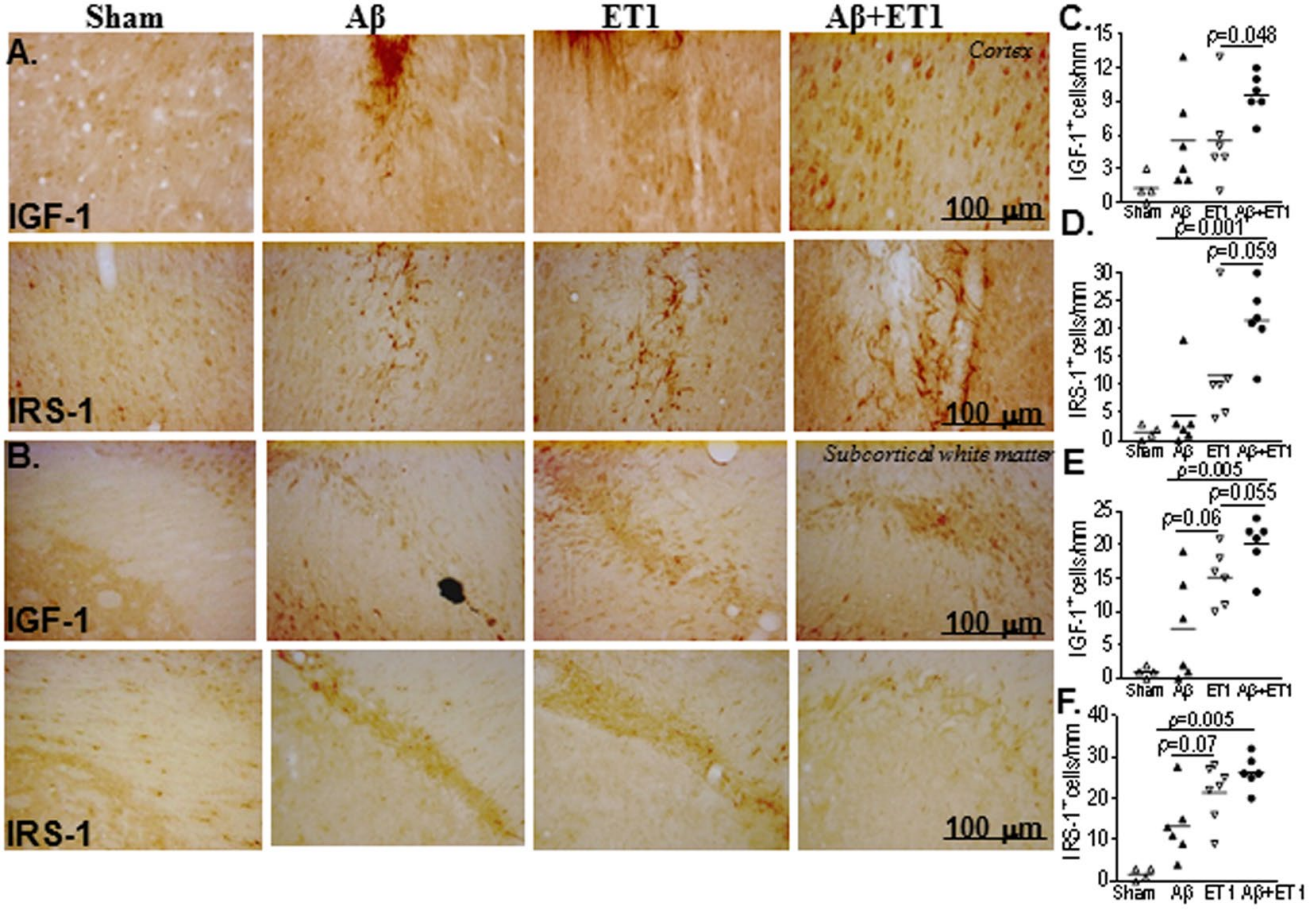
Cortex and subcortical white matter: High resolution immunostaining indicates staining for IGF-1 and IRS-1 in the ipsilateral cortex (**A**) and subcortical white matter (**B**) of sham, Aβ, ET1 and Aβ+ET1 rats. Plots show quantitative assessment of IGF-1 and IRS-1 staining in the cortex (**C** and **D**) and subcortical white matter (**E** and **F**) of sham, Aβ, ET1 and Aβ + ET1 rats.

Assessment of the motor cortex revealed a borderline increase in the leakage of IgG as well as the presence of IGF-1 and IRS-1positive cells in the vicinity of the site of injections in Aβ + ET1 rats when compared to the ET1 (IGF-1 *p* = 0.048, IRS-1*p* = 0.059) and Aβ (IGF-1 *p* = 0.059, IRS-1*p* = 0.001) rats (Fig. 2A,C,D). IRS-1 positive cells were also evident in the ventral cerebral cortex of all 3 experimental groups at the level of the insular cortex. Subcortical white matter, which is essentially devoid of neuronal cell bodies, was seen filled with astrocytic cells in Aβ, ET1 and Aβ + ET1 rats (Fig. 2B). The Aβ + ET1 interventions resulted in a general increase in both IGF-1 and IRS-1 stained cells compared to ET1 (IGF-1 *p* = 0.055) and significantly more than Aβ (IGF-1 *p* = 0.005, IRS-1*p* = 0.005) rats (Fig. 2B,E,F) that from morphology and appearance look like astrocytes (Fig. 2B). Astrocytes are also reported to express significantly higher levels of IRS-1^28^. Moreover, Aβ and Aβ + ET1 rats also demonstrated a bilateral (*contralateral not shown*) increase in the number of astrocytes compared to unilateral increase in ET1 rats.

### Hippocampus and septohippocampus

**Figure 3:**
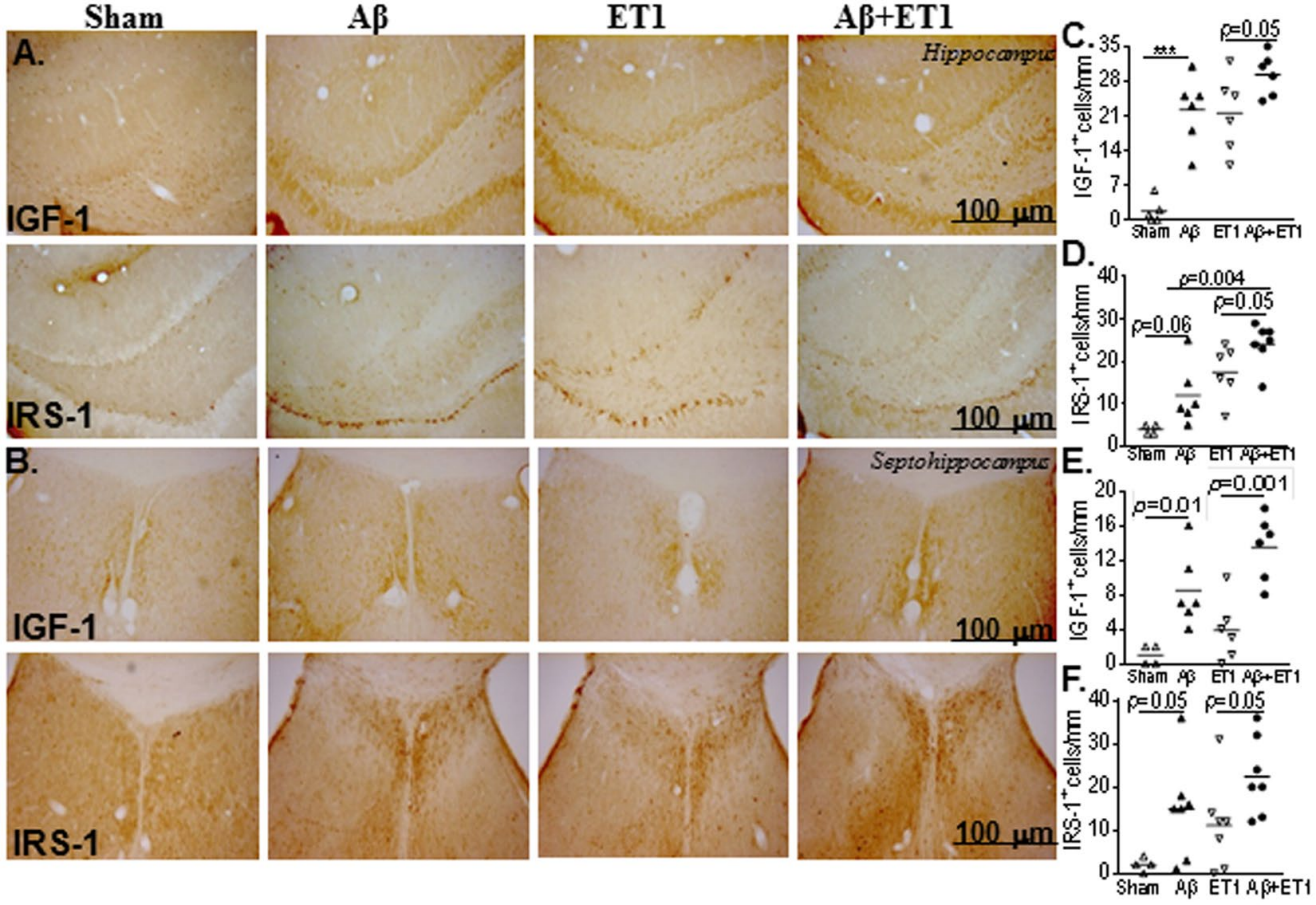
Hippocampus and septohippocampus: High resolution immunostaining indicates staining for IGF-1 and IRS-1 in the ipsilateral dentate gyrus of hippocampus (**A**) and the septohippocampus (**B**) of sham, Aβ, ET1 and Aβ + ET1 rats. Plots show quantitative assessment of IGF-1 and IRS-1 staining in the dentate gyrus (**C** and **D**) and septohippocampus (**E** and **F**) of sham, Aβ, ET1 and Aβ + ET1 rats, *p < 0.05, **p < 0.01.

The number of IGF-1 and IRS-1 positive cells which appeared in the ipsilateral dentate gyrus of Aβ + ET1 rats were substantially more compared to ET1 (IGF-1 *p* = 0.05, IRS-1 *p* = 0.05) and significantly more than Aβ (IRS-1 *p* = 0.004) rats (Fig. 3A,C,D). Aβ + ET1 rats also showed increased IGF-1 and IRS-1 staining in the contralateral (*not shown*) dentate gyrus. The septohippocampus demonstrated a significantly increased number of IGF-1 and IRS-1 positive cells in Aβ and Aβ + ET1 rats compared to sham (IGF-1 *p* = 0.01, IRS-1 *p* = 0.05) and ET1 (IGF-1 *p* = 0.001, IRS-1 *p* = 0.05) brains, respectively (Fig. 3B,E,F). Interestingly, the IGF-1 positive cells were observed in the vicinity of microvessels, whereas IRS-1 antibody showed deposition of Aβ-like fragments around microvessels in cells that from appearance look like neurons and astrocytes.

### Periventricles and basal forebrain

**Figure 4:**
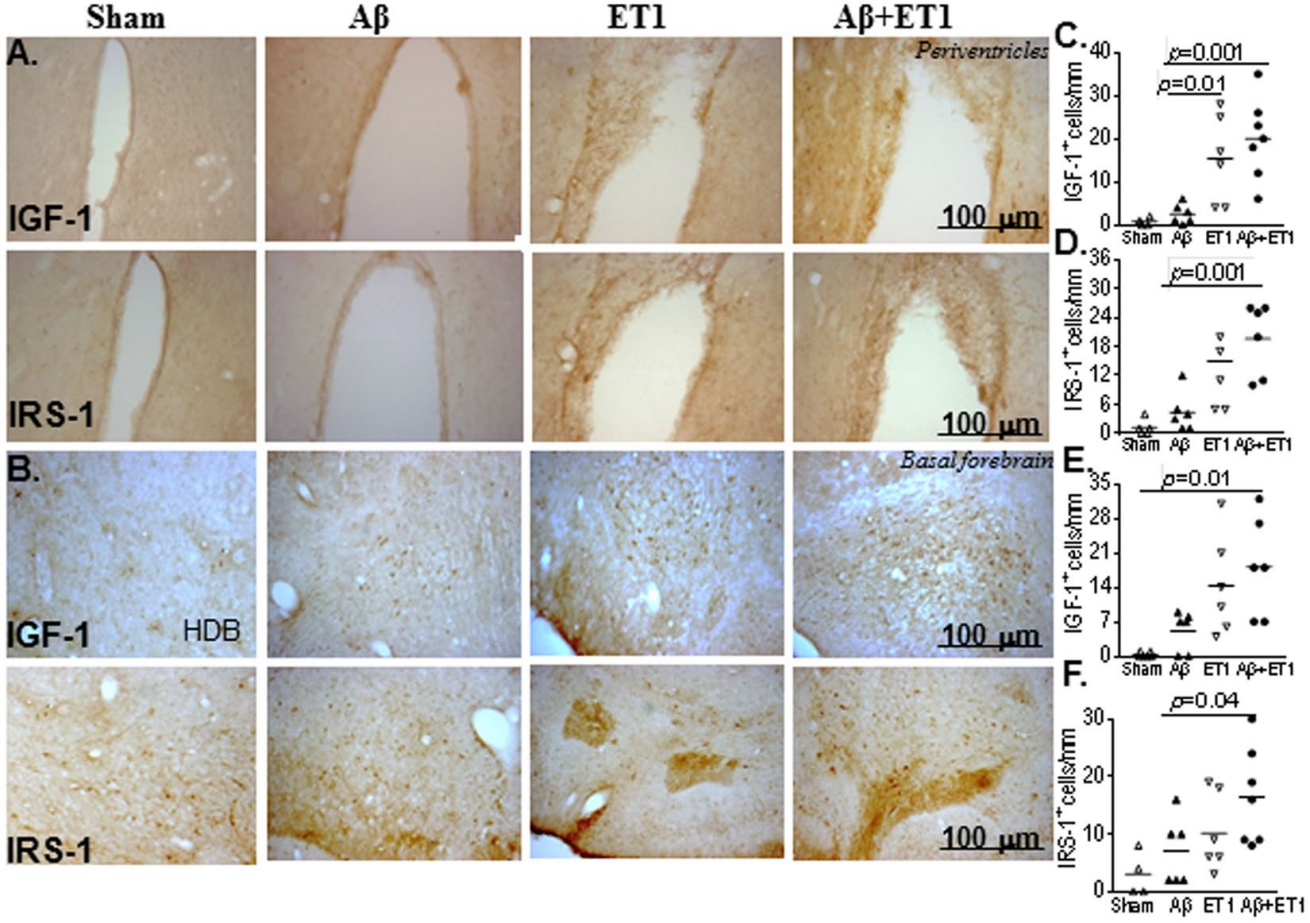
Periventricles and basal forebrain: High resolution immunostaining indicates staining for IGF-1 and IRS-1 in the ipsilateral periventricles (**A**) and horizontal diagonal band (HDB) of Broca in basal forebrain (**B**) of sham, Aβ, ET1 and Aβ + ET1 rats. Plots show quantitative assessment of IGF-1 and IRS-1 staining in the periventricles (**C** and **D**) and HDB (**E** and **F**) of sham, Aβ, ET1 and Aβ + ET1 rats.

Additional brain areas observed to have pathological changes including periventricular regions either superior to the anterior ipsilateral caudate putamen or in areas close to the anterior horns of both lateral ventricles with significantly more IGF-1 (*p* = 0.001) and IRS-1 (*p* = 0.001) cells in Aβ + ET1 rats compared to Aβ rats (Fig. 4A,C,D), respectively. This also included ventricle enlargement in Aβ, ET1 and Aβ + ET1 rat brains three weeks after the surgery, strengthening our previous findings^6^. IGF-1 and IRS-1 staining also prominently increased in the contralateral periventricular region (*not shown*) of Aβ and Aβ + ET1 rats. The horizontal diagonal band (HDB) of Broca in the basal forebrain demonstrated an increased tendency of IGF-1 and IRS-1 positive cells in ET1 and Aβ + ET1 rats compared to sham and Aβ brains (Fig. 4B,E,F); however, it was not statistically significant.

## Discussion

Previously we have described various important pathological alterations in co-morbid models of Aβ toxicity and ischemia in both non-transgenic rats and transgenic mice^5,6,25^. In the present study we have undertaken a more detailed analysis of the insulin and IGF-1 signaling pathway to dissociate the synergistic or additive connection between the early pathological events of dementia, such as high levels of Aβ and neuroinflammation, and the small focal ischemia. Here we showed that Aβ toxicity and ischemia provoked substantial region-specific increases in the abundance of cells staining for IGF-1 and IRS-1 that encompassed the majority of the ipsilateral hemisphere. This is the first study to demonstrate an overall upregulation in IGF-1 and IRS-1 presence after co-morbid occurrence of Aβ toxicity and striatal infarct.

Focal cortical ischemia has been shown to evoke inflammatory responses in the perilesional areas. In the present study, although the striatum and thalamus were the predominant damaged regions in ET1 and Aβ + ET1 rats, histological alterations were also noticed in distant regions^29^ with synaptic links to the striatal and thalamic lesions, such as subcortical white matter, motor cortex, septohippocampal nucleus, dentate gyrus, periventricular regions and HDB. Conversely, the regional injury induced by ET1 or Aβ toxicity alone was not severe enough to significantly affect the entire brain circuit as reported by us recently^5^.

An increase in the number of IGF-1 or IRS-1 immunoreactive cells in the cortex again implies the potential critical importance of this region in the cascade of events for the interactions between ischemia and Aβ toxicity^5^. Importantly, due to the massive neurodegeneration in the associative cortices, AD was formerly referred to as cortical dementia^30^. The presence of IGF-1 and IRS-1 positive cells around the temporal horns of the lateral ventricles, which are close to the paralimbic region, arguably suggests a role for insulin signaling in cognitive performance in Aβ + ET1 rats. This region is particularly vulnerable during the prodromal stages of dementia^31^. Furthermore, an investigation of surface map changes in the temporal horns of AD patients correlated regional enlargement of lateral ventricles to the progression of disease^32^. Similarly, AD pathology in HDB, relative to rest of the brain, might imply a greater vulnerability of the neurons in the HDB to plaque pathogenesis, possibly due to the impaired neurogenesis in the nearby olfactory piriform. The neurogenic potential (though not as robust as in hippocampus and the subventricular zone) of layer II of the piriform cortex in neuronal differentiation of newborn cells of adult rats has been well characterized^33^.

Intriguingly, like Aβ + ET1 rats, lowered sensory cortico-cortical and the thalamo-cortical potential has also been reported in the diabetic rat brain^34^. Perhaps, direct connections of the thalamus to the hippocampus (through the fornix) might explain the increased number of IGF-1 and IRS-1 positive cells in the dentate gyri of Aβ, ET1 and Aβ + ET1 rats. Few or reduced processes identified in IGF-1- or IRS-1-positive neurons in the thalamus agrees with the observation of reduced number of dendrites in the diabetic rat brain^34^.

An acute increase in IGF-1 post-surgery could reflect possibly either a reparative or protective response by the damaged neuronal cells^35–37^ or an increased accumulation/synthesis of IGF-I or enhanced input of IGF-I from peripheral resources (reactive glial cells) - to enhance their availability to the injured region^38^. It has been reported that neurons may become IGF-I resistant in regions going through an inflammatory process due to the actions of pro-inflammatory cytokines such as tumor necrosis factor (TNF) α.TNFα interferes with insulin/IGF-I receptor coupling to attenuate insulin/IGF-I signaling^17^ as well as by inducing IRS-1 phosphorylation^39^, possibly via reducing c-Jun N-terminal kinase (JNK) activation^40^, which may increase the levels of IGF-1^38^. This might be the case in Aβ + ET1 rats, which exhibit significantly higher inflammation^5,6^. Knockdown studies of IGF-1R/IR signaling in *Caenorhabditis elegans* demonstrating reduction in aggregation-mediated Aβ1–42 toxicity^41^ not only provides a direct relationship between IGF-1R/IR signaling and Aβ toxicity but could also explain the increased Aβ production^5^ and FJB positive cellular degeneration^5^ in our Aβ + ET1 rats^5,6^ with the concurrent increase in IGF-1 and IRS-1 expression in the present study.

Computed tomography scans and magnetic resonance imaging have commonly identified lesions in subcortical white matter of demented and elderly patients, as hypertensive territories^42,43^; however, astrocytic IGF-I (and IRS-1) expression in subcortical white matter might be protective as IGF-I-related peptides, when expressed by astrocytes, may reduce immune-mediated myelin injury during lesion progression and recovery^44^, and recovery from insults such as hypoxia–ischemia (*reviewed in*^45^. This also provides an explanation for the restoration of the blood-brain barrier in the ET1 rats observed by us recently^46–48^. Likewise, IRS-1 deficits have been shown to contribute to insulin resistance in animal models and diabetic patients^49^.

Our research may be relevant to the linkage between insulin signaling and inflammatory processes in a vulnerable brain. The present study addressed the regional differences in the IGF-1 and IRS-1 expression in response to ischemia and high amyloid toxicity, enabling us to recognize the brain regions that are not only sensitive to insulin signaling but can also be considered important to VCI prevention. This approach may eventually lead to the future clinical strategies to interrupt the mechanisms mediating the effects of vascular risk factors on cognitive decline^7,50^. Further, an investigation into the IRS-1 residues that are phosphorylated by the co-morbid injury might be an interesting future investigation implying its activation or inhibition to bind to the receptor to subsequently down or upregulate the insulin signaling. As, serine phosphorylation of IRS-1 has been reported as an important feature in AD brain resulting in the failure of IRS-1 to transmit insulin receptor signals to the downstream signaling machinery^51^.

## Materials and Methods

### Animal, treatment and tissue preparation

All animal protocols were carried out according to the guidelines of the Animal Care and Use Committee of Western University (approval ID: 2008-113) and NIH. All animal protocols were approved by Animal Care and Use Committee of Western University. Male Wistar rats (Charles River, Montreal QC, Canada, 250 to 310 gm) were anesthetized using sodium pentobarbital (60 mg/kg, i.p, Ceva, Sante Animale). The animals were positioned in a stereotaxic apparatus (David Kopf) with the incisor bar below the interaural line, set at 3.3 mm. Body temperatures were maintained at 37 °C. To insert the cannula (30 gauge), small burr holes were drilled in the parietal bone. Four groups of animals were studied (n = 4–7 for each group). To model striatal ischemia (**ET1 group**) a single injection of 6 pmol endothelin-1 (ET1; Sigma-Aldrich, Oakville, ON) per 3 µL (dissolved in saline) was made into the right striatum as described^5,6^ through the cannula (anterior/posterior + 0.5 mm, medial/lateral -3.0 mm relative to bregma, and dorsoventral –5.0 mm below dura). The rat model of β-amyloid toxicity (**A**β **group**) was produced by intracerebroventricular (ICV) non-aggregated Aβ25-35 injections as described by us elsewhere^5,6,25^. Briefly, Aβ25-35 peptide (Bachem, Torrance, California) at 50 nmol/10 µL dissolved in saline was injected into the lateral ventricles bilaterally (anterior/posterior: –0.8 mm, mediolateral: ±1.4 mm relative to bregma, and dorsoventral: −4.0 mm below dura). The toxic fragment, Aβ25-35, has been shown in AD brains^52,53^, and in *in vivo* and *in vitro* investigations^52^ to elicit neuronal degeneration, neuroinflammation with reactive astrocytosis and functional impairments (*reviewed in* Kaminsky *et al*.^54^). An additional benefit of using Aβ25-35 is to induce modest pathological alterations that can be combined with a minor ischemia model to study the interactions. For rats receiving both bilateral intracerebroventricular (ICV) Aβ25-35 injections and unilateral striatal ET1 injections (**A**β** + ET1 group**), the Aβ25-35 peptide injection into the lateral ventricles was followed by the ET1 injection into the striatum. The sham-treated rats (**Sham group**) received all the surgical steps without injections of Aβ25-35, or ET1. It has been proven in the earlier studies that control rats receiving reverse scrambled peptide Aβ 35-25 do not show any pathology, either alone or when combined with endothelin-1^55^. The Paxinos and Watson atlas was used to determine all stereotaxic coordinates^56^. Following ET1 or Aβ25-35 injections, the syringe was left *in situ* for 3 minutes before being removed slowly. After suturing the wound all rats received subcutaneous injection of 30 µg/kg buprenorphine and an intramuscular injection of 20 μl (50 mg/ml stock) enrofloxacin antibiotic (Baytril, Bayer Inc., Canada), and were subsequently allowed to recover from surgery. Three weeks after surgery animals were euthanized with 160 mg/kg of pentobarbital by i.p. injection and transaortically perfused with 4% paraformaldehyde (pH 7.4). The brains were removed, post-fixed in 4% paraformaldehyde for 24 h, and cryoprotected by immersion in 30% sucrose for 36 hours at 4 °C.

### Histology

Immunohistochemistry was performed on serial, coronal cryo-sections of the entire brain, 40 µm in thickness (using a sliding microtome, Tissue-Tek Cryo3, USA), with primary antibodies against major histocompatibility complex class II antigen produced by microglia (OX-6, BD Pharmingen, 554926, 1:1000), amyloid precursor protein (APP), Aβ and its 17–24 fragment (4G8, Signet, Covance, Emeryville, CA, USA, 9220-10) and FluoroJade B (FJB; Chemicon Int., 0.0004%) to examine the cellular degeneration, IGF-1 (Santa Cruz, Sc-9013, 1:500) or IRS-1 (Upstate Cell Signaling Solution, 06-248, 1:500) and secondary antibodies horse anti-mouse (BA-2000) and goat anti-rabbit (BA-1000) as described elsewhere^6^^,57^. In all cases, secondary antibodies, serum and ABC reagent were from the Vectastain Elite ABC Kit (Vector Laboratories, Inc., Burlingame, CA, USA). Fluorochrome FJB staining is described elsewhere^5^.

### Analyses

All data analyses were performed blinded and with adequate allocation concealment. Light microscopy was used to carry out the histological analyses of brain sections. Images were taken with a Leica Digital Camera (DC 300, Leica Microsystems Ltd., Heerbrugg, Switzerland) attached to a Leitz Diaplan Microscope. Digitized images acquired using 10× objective were saved as TIFF files with an identical level of sharpness and contrast using the IM50 software. Six randomly chosen fields on region of interest on 6 non-neighboring sections were studied from each brain, taken from the anterior to posterior levels (1.7 to −3.14 mm from the bregma or altogether 4.84 mm) with intervals of 240 µm each. Cellular densities on each of the six slices (expressed as the number of stained cell tops per mm in the optical field) were calculated as the arithmetic mean number of cells divided by the total area of the region analyzed in the ipsilateral hemispheres of each animal. The results were displayed as the numbers of stained cells per mm^2^ of each region analyzed.

### Statistical Analyses

All values were presented as mean ± standard error of the mean (S.E.M.). Number of IGF-1 and IRS-1 labeled cells, were analyzed using parametric unpaired ttest that assumes equal distribution and one-way ANOVA followed by *post hoc* Dunnett tests using GraphPad Prisim version 5.0 for Windows (La Jolla California USA). The significance level was *p* ≤ 0.05.
